# Split Ends Inhibits the Dedifferentiation of imINP to Prevent the Generation of Supernumerary Type II Neuroblasts in *Drosophila*

**DOI:** 10.3390/cells14231926

**Published:** 2025-12-04

**Authors:** Qingxia Zhou, Fuhao Zhang, Sifan Gong, Shuliu Zhang, Wenting Gong, Menglong Rui, Su Wang

**Affiliations:** 1 Key Laboratory of Development Genes and Human Disease, Institute of Microphysiological Systems, School of Life Science and Technology, Southeast University, Nanjing 210096, China; 230218974@seu.edu.cn (Q.Z.); zhang-fuhao@seu.edu.cn (F.Z.); 230208387@seu.edu.cn (S.G.); 230208386@seu.edu.cn (S.Z.); 220223549@seu.edu.cn (W.G.); 2 Co-Innovation Center of Neuroregeneration, Nantong University, Nantong 226019, China

**Keywords:** type II NBs, *spen*, imINP dedifferentiation, Notch pathway, *Hairless*

## Abstract

Investigating the mechanisms that maintain different types of neural stem cells is essential for brain development. While factors maintaining distinct *Drosophila melanogaster* neuroblasts (NBs) have been identified, additional factors remain unidentified. In this paper, we find knockdown of *split ends* (*spen*) increases in nuclear Notch intracellular domain (NICD) level, which in turn activates Notch signaling in type II NBs. This activation causes the intermediate neural progenitors (imINPs) to dedifferentiate into type II NBs, thereby increasing the number of type II NBs specifically. Additionally, we find that knockdown of both *spen* and a co-repressor of the Notch signaling pathway, Hairless, in type II NBs exacerbates the increase in type II NBs number, compared to *spen* knockdown alone. Furthermore, we observe that loss of Spen results in more severe phenotypes than loss of Hairless in type II NBs and their lineages. We reveal that Spen may indeed function as a functional homolog of its mammalian homolog, SHARP, acting as a novel Notch signaling co-repressor in type II NBs specifically. This highlights the potential for multiple co-repressors to collaboratively regulate the same signaling pathway within the type II NBs lineage. The distinct regulatory mechanism of type I and II NBs offers new insights into the study of neural stem cell homeostasis.

## 1. Introduction

Different regulatory mechanisms are crucial for the maintenance of distinct NBs and are also important for the overall homeostasis of brain development. Neural stem cells are a type of cell that generate neurons or glial cells, which compose the central nervous system (CNS) [1,2]. Excessive proliferation of neural stem cells may induce the onset of malignancies, whereas insufficient division of neural stem cells can lead to neurodevelopmental defects [3,4]. Mammalian neural stem cells exhibit various division modes, and the division modes of type I and type II neural stem cells (neuroblasts, NBs) [5] in *Drosophila melanogaster* are analogous to two of these modes in mammals [1,6]. Therefore, *Drosophila* NBs serve as an excellent model for studying neural stem cells. The *Drosophila* central brain NBs are primarily categorized into two types: type I and type II NBs [7]. *Drosophila* NBs originate during embryogenesis, and by the third instar larval stage, there are eight type II NBs per hemisphere, a number that is significantly smaller than the approximately 90 type I NBs [8,9]. In addition, the progeny cells produced by the two types of NBs differ. Type II NBs generate another NB and an intermediate neural progenitor (INP), which undergoes a limited number of division cycles before differentiating into a ganglion mother cell (GMC), while type I NBs give rise to another NB and a ganglion mother cell (GMC), which subsequently produces neurons or glial cells [1,7]. Different types of neural stem cells produce varying numbers of progeny and contribute to distinct brain structures [10,11,12,13]. For example, type II NBs can generate numerous progeny cells that contribute to the formation of the central complex of the *Drosophila* central brain, or to the optic lobe by producing glial cells which differentiate into lobular giant glial cells [1,8,9,10,12]. In addition to the aforementioned differences, type I and type II NBs also express distinct molecular markers. Asense (Ase) is specifically expressed in type I NBs, but not in type II NBs, whereas Pntp1 (Pnt) is exclusively expressed in type II NBs [14,15]. Many studies have reported the different maintenance mechanisms of type I and type II NBs. For example, *Six4* can specifically inhibit the premature differentiation of INPs [16]. However, it remains unclear whether there are other unknown specific regulatory factors that affect different types of NBs. The process by which type II NBs generate INPs is analogous to that of higher mammalian NSCs, making type II NBs an excellent model for studying the maintenance of neural stem cells [17,18]. Therefore, it is crucial to explore the mechanisms involved in maintaining *Drosophila* type II NBs specifically.

It has been reported that ectopic activation of Notch signaling leads to over-proliferation and an increase in ectopic NBs, which appears to be more pronounced in type II NBs [19,20,21,22]. The mechanism by which the Notch signaling pathway exerts its effects through cleavage is conserved. Upon binding of the Notch receptor to ligands secreted by adjacent cells, a series of cleavage events occurs, resulting in the generation of the active form, Notch^NICD^ (Notch intracellular domain, NICD). Notch^NICD^ subsequently translocates from the cytoplasm to the nucleus, where it activates the expression of downstream Notch target genes such as E(spl)mγ [23,24]. During the activation of the Notch signaling pathway, numerous factors regulate this process. For example, the mammalian SHARP functions as a co-repressor recruited by RBP-J. In the absence of Notch^NICD^, SHARP and RBP-J bind to DNA, thereby repressing the expression of downstream target genes [24,25,26]. This process is mediated by Hairless (H) in *Drosophila*; however, no homologous protein of Hairless has been identified in mammals [24,27]. Although many regulatory factors have been reported, it remains unclear whether there are other regulatory factors of the Notch signaling pathway and whether multiple co-repressors exist to coordinately regulate the Notch signaling pathway in *Drosophila* type II NBs. Therefore, investigating new regulatory factors of the Notch signaling pathway is crucial for the maintenance of type II NBs.

Split ends (Spen) (also called MINT in mice and SHARP in humans [28,29]) play an important role in regulating gene expression and tissue development. SHARP, as a transcriptional co-repressor, can combine with chromatin-remolding complexes or physically associate with the nuclear receptor components [30,31,32,33,34]. The biological functions of Spen include promoting cilia formation, maintaining middle glial cell fate, and regulating various other processes [35,36,37]. In addition, Spen is involved in multiple signaling pathways that collectively regulate tissue growth and development [38,39,40,41]. For example, during *Drosophila* eye development, the absence of Spen results in ectopic activation of Notch signaling, and this aberrant activation subsequently diminishes the activity of the epidermal growth factor receptor (EGFR) signaling pathway, ultimately leading to the disruption of adult eye morphology [41]. However, the role of Spen in *Drosophila* NBs is currently unclear, and the relationship between the Notch signaling pathway in *Drosophila* type II NBs remains to be elucidated. Furthermore, it remains unclear whether multiple signaling pathways collaborate to exert their effects in *Drosophila* NBs and whether Spen plays a critical regulatory role in NBs.

In this study, we find that Spen can prevent the generation of supernumerary type II NBs but does not affect the number of type I NBs. Moreover, we identify that the specific role of Spen in type II NBs is mediated by the inhibition of the Notch signaling pathway, which prevents the dedifferentiation of intermediate neural progenitors (imINPs). In addition, we also find that knockdown of both *spen* and *Hairless* can enhance the phenotype resulting from knockdown of *spen* alone in type II NBs. Furthermore, in imINPs, Hairless and Spen appear to play distinct roles. The reduction in the EGFR signaling pathway can partially rescue the increase in type II NBs caused by *spen* knockdown. Therefore, our experiments highlight that Spen functions as a novel regulatory factor of Notch signaling in *Drosophila* to prevent the generation of supernumerary type II NBs specifically. This regulatory role suggests that Spen, like Hairless, may function as an ortholog of mammalian SHARP.

## 2. Materials and Methods

### 2.1. Drosophila Stocks and Genetics

Flies were raised at 25 °C and mated at 29 °C. The GAL4 strains involved in this paper included: UAS-Dicer2; wor-GAL4, ase-GAL80, UAS-mCD8-GFP; + (II NB-GAL4), w; ase-GAL4; UAS-Dicer2, w; UAS-Dicer2; PntP1-GAL4, UAS-mCD8-GFP (Pnt NB-GAL4), w; UAS-mCD8-GFP; UAS-Dicer2, 9D11-GAL4 (9D11 mINP-GAL4), w; UAS-LacZ, 9D10-GAL4 (9D10 mINP-GAL4); sb/Tm6B, Repo-GAL4/Tm6B, Elav-GAL4, Spen-GAL4, Hairless-GAL4. The other strains involved in this paper included: UAS-*spen* RNAi (Tsing Hua Fly Center, Beijng, China, THU0750), UAS-*spen* RNAi (Vienna Drosophila Resource Center (VDRC), Vienna, Austria, 108828, P{KK100153}VIE-260B), UAS-*spen* RNAi (v48846, gift from Li Hua Jin), UAS-*spen* (Bloomington Drosophila Stock Center (BDSC), Bloomington, Indiana, USA, 20756,y1 w67c23; P{EPgy2}*spen*EY12567), Pnt-lacZ (gift from Zhouhua Li), UAS-luciferase (BDSC35788, P{UAS-LUC.VALIUM10}attP2), UAS-LacZ RNAi (V51446), UAS-*Notch* RNAi (THU0549), UAS-*Notch* RNAi (BDSC33611, P{TRiP.HMS00001}attP2), w; +; E(spl)mγ-GFP (gift from Yan Song), UAS-*Egfr* RNAi (THU1863, THU1864), UAS-*Egfr* CA (BDSC9533, BDSC9534), UAS-*Hairless* RNAi (THU3690), UAS-*Hairless* RNAi (v24466), UAS-*Arm* RNAi (THU1631), UAS-*Ctbp* RNAi (THU1078), UAS-*Ctbp* RNAi (THU1919), UAS-*brat* (BDSC13860).

### 2.2. Immunohistochemistry

Third larval brains were dissected, and then they were incubated in 4% paraformaldehyde for 20 min. Samples were washed with 0.3% PBST for 4 times and subsequently blocked by 2% BSA for 1 h. Samples were incubated at 4 °C overnight with primary antibodies. The samples were washed with 0.3% PBST for 4 times, then the secondary antibody was incubated for 2 h, and the secondary antibody was finally washed off. Paraformaldehyde (Sigma, Merck Ltd., Shanghai, China). PBST is prepared by adding TritonX-100 to PBS (Phosphate-Buffered Saline). TritonX-100 (Shanghai Shenggong Biotechnology Engineering Co., Ltd., Shanghai, China). PBS (Shanghai Shenggong Biotechnology Engineering Co., Ltd., Shanghai, China). Finally, the tissues were observed with Zeiss LSM700 (Carl Zeiss Microscopy GmbH 700, version 3.6, Zeiss, Germany, Jena) and Zeiss LSM900 (Carl Zeiss Microscopy GmbH 900, version 3.8, Zeiss, Germany, Jena) confocal microscopes. The following primary antibodies were used in this paper: Chicken polyclonal anti-GFP (1:1000, Cat# A10262, Thermo Fisher Scientific, Shanghai, China), Rat monoclonal anti-Miranda (1:1000, Cat#ab197788, Abcam, Shanghai, China), Rat monoclonal anti-Dpn (1:1000, Cat# ab195173; Abcam), Rabbit polyclonal anti-PH3 (1:100, Cat# 9701, Cell Signaling Technology, Shanghai, China), Rat anti-Elav (1:50, Cat# 9F8A9, DSHB (Developmental Studies Hybridoma Bank), Shanghai, China), Rabbit anti-Ase (Serum antibodies constructed by the laboratory), Mouse anti-PKC ζ (1:50, Cat#177781, Santa Cruz, Shanghai, China), Chicken anti-lacZ (1:20, Cat#ab9361, Abcam), Mouse anti-NICD (1:50, cat#C17.9C6, DSHB). Mouse anti-NECD (1:50, cat#C458.2H, DSHB), Rabbit anti-mcherry (1:10, Cat#ab213511, Abcam).

### 2.3. The NICD Level Analysis

During the microscope scanning, continuous observation and scanning along the *Z*-axis are performed, and the layers with the maximum cross-section on the two-dimensional plane of the entire cell for imaging are selected. Then the single-layered nucleus identified in the 2D images is the region of interest for further analysis of NICD levels. Fold changes in mean fluorescence intensities of NICD are represented in the quantitative fluorescence figures.

### 2.4. Statistical Analysis

For quantification of NSCs, Dpn/Mira and GFP-positive NSCs at the indicated stage were counted. Fluorescence intensity analysis was performed on samples under consistent background conditions using ImageJ software (version Fiji Is Just ImageJ 2.9.0/1.53t). Fluorescence intensity and other statistical data were analyzed using GraphPad Prism 6 (GraphPad Software). For comparisons between two groups, the *t*-test or the non-parametric Mann–Whitney Test was employed to assess statistical significance. The total number of animals, analytical methods, *p*-values, and significance levels were indicated in the Figure legends. *p*-values of less than 0.05 were considered statistically significant. Asterisks indicate critical levels of significance (*: *p* < 0.05; **: *p* < 0.01; ***: *p* < 0.001; ****: *p* < 0.0001). Table S1 shows the exact *p*-values of this article.

## 3. Results

### 3.1. Spen Knockdown Leads to an Increased Number of Type II NBs Specifically

**Figure 1:**
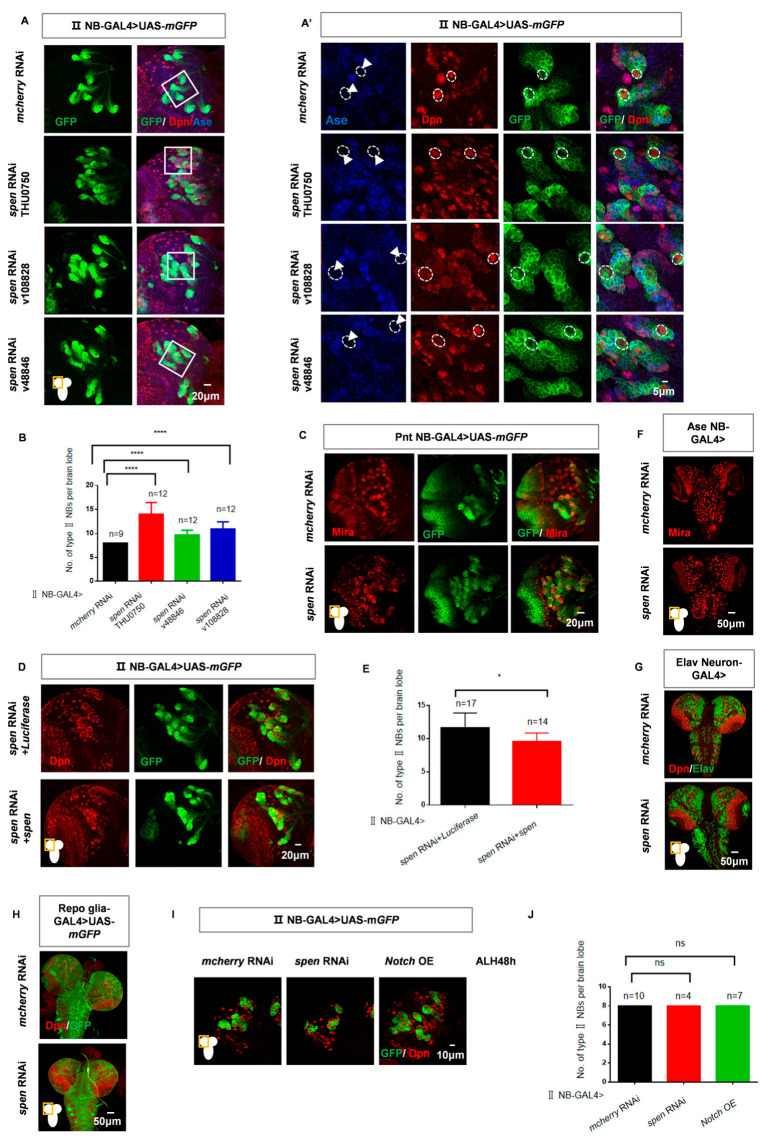
*split ends(spen)* knockdown leads to an increased number of type II neuroblasts(NBs) specifically. (**A**) Utilizing type II NB-GAL4 to knock down different *spen* RNAi lines consistently resulted in an increase in type II NBs. The area outlined by the white solid line represents the region that was magnified for scanning. (**A’**) showed type II NB lineages, e labeled with GFP, Dpn, but not Ase (white arrowhead) in (**A**). The white dashed circles represent type II NBs. (**B**) Quantification of type II NBs number in per brain lobe about (**A**). **** *p* < 0.0001, Mann–Whitney Test for analysis. (**C**) Knockdown of *spen* by Pnt-GAL4 also induced an increased number of type II NBs. (**D**,**E**) Overexpression of *spen* in a background where *spen* was knocked down can partially rescue the number of ectopic type II NBs, and (**E**) shows quantification of the number of type II NBs in each brain lobe. * *p* < 0.05, Mann–Whitney Test for analysis. (**F**–**H**) Knockdown of *spen* in type I NBs by Ase-GAL4 (**F**) and in neurons by Elav-GAL4 (**G**) and in pan-glial cells by Repo-GAL4 (**H**) resulted in no obvious effect on the whole brain size and NBs. (**I**,**J**) At ALH48h, the number of type II NBs had no obvious change in *spen* knockdown and Notch overexpressing flies. (**J**) Quantification of type II NBs number in per brain lobe from genotypes in (**I**), Mean ± SEM, ns, non-significant, *t*-test for analysis. Mira or Dpn represented NBs in all results. Elav showed neuronal cells in (**G**), and GFP showed the structure of glial cells in (**H**). All brains were obtained from third-instar larvae. Type II NB lineages are labeled by GFP and Dpn.

In order to identify genes that specifically influence the development of type II NBs, we conducted knockdown experiments using a GAL4 driver that was specific to type II NBs (UAS-Dicer2; wor-GAL4, ase-GAL80, UASmCD8-GFP, referred to as II NB-GAL4 hereafter). Then, we quantified the number of type II NBs in each brain hemisphere (in wildtype, type II NBs can be labeled with Dpn and GFP, but not with Ase) at the third instar larval stage. We found that *spen* knockdown resulted in a greater number of type II NBs compared to control (Figure 1A,B). Increased numbers of type II NBs were also observed in additional *spen* RNAi lines (Figure 1A,B). Among these strains, the THU0750 strain was selected for the subsequent experiments. Simultaneously, we employed a different type II NB-GAL4 (UAS-Dicer2; Pnt-GAL4, UAS-mCD8-GFP, hereafter referred to as Pnt-GAL4) for *spen* knockdown and also found an increase in the number of type II NBs (Figure 1C). To rule out the possibility of off-target effects of RNAi, we performed a rescue experiment by overexpressing *spen* in a background where *spen* was knocked down. We found that knockdown of *spen* followed by overexpression of *spen* can reduce the increased number of type II NBs induced by *spen* knockdown (9.6 NBs compared to 11.6 NBs) (Figure 1D,E). These results demonstrate that the phenotype of type II NBs number increase is indeed caused by *spen* knockdown.

To investigate whether this phenotype was specific to type II NBs, we utilized additional GAL4 drivers to knock down *spen* in other tissues. There is no obvious alteration in the number of type I NBs when *spen* was knocked down by Ase-GAL4 (Figure 1F). Furthermore, knockdown of *spen* in neurons by Elav-GAL4 or glial cells by Repo-GAL4 (Repo-GAL4; UAS mCD8-GFP, referred to as Repo-GAL4) did not cause any obvious phenotype (Figure 1G,H). These results suggest that knockdown of *spen* increases the number of type II NBs specifically.

Finally, we investigated the time period during which Spen exerts its effects. By quantifying the number of type II NBs at 48 h after larval hatching (ALH), we found that there is no difference in the number of type II NBs when *spen* knockdown at ALH48h compared to control (Figure 1I,J). It demonstrates that Spen plays a role at the late second instar larval stage and thereafter. These experimental results reveal that knockdown of *spen* specifically increases the number of type II NBs at the late stage of second instar larva and thereafter.

### 3.2. Spen Prevents Type II NBs Number Increase Excessively by Inhibiting the Dedifferentiation of ImINPs

To explore the causes of the increase in the number of type II NBs, we first detected the asymmetric divisions. Disruption of asymmetric division can impact the self-renewal capacity of type II NBs, thereby altering their overall numbers [42]. Proper orientation of cell fate determinants is essential during asymmetric division. The Par complex, which includes components such as aPKC, localizes to the apical cortex of NBs and is ultimately distributed to the larger daughter cell, forming a new NB. Conversely, factors like Miranda (Mira) localize to the basal cortex and are allocated to smaller progeny cells [1,43,44]. So, we detected the location of Mira and aPKC in the metaphase with *spen* knockdown. We observed that aPKC was retained apically within the NBs, while Mira is localized at the basal cortex of the NBs, adjacent to the newly generated INPs (Figure S1A,B). Consequently, these results suggest that *spen* knockdown does not result in asymmetric division defects.

**Figure 2:**
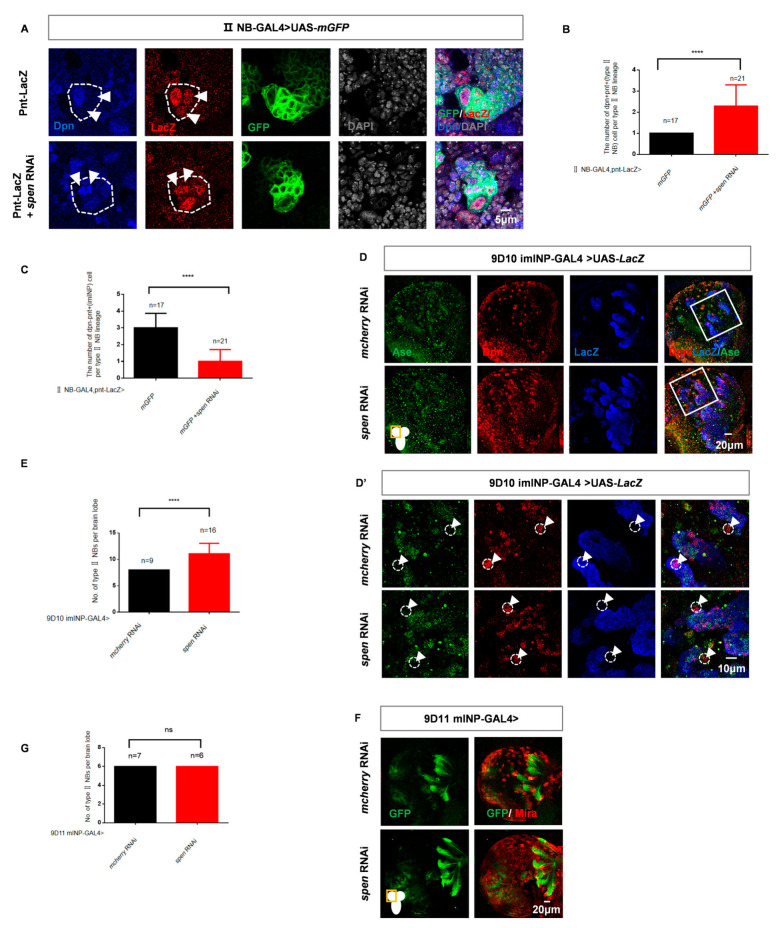
*spen* knockdown induces intermediate neural progenitors (imINPs) dedifferentiate into type II NBs. (**A**–**C**) *spen* knockdown induced the Dpn+ Pnt+ (NB) cells number increase (white arrowhead), and Dpn- Pnt+ (imINP) cells decrease. (**B**) Quantification of Dpn+ Pnt+ (NB) cells number per II NB lineage from genotypes in (**A**). **** *p* < 0.0001, Mann–Whitney Test for analysis. (**C**) Quantification of Dpn- Pnt+ (imINP) cells number per II NB lineage from genotypes in (**A**). Mean ± SEM, **** *p* < 0.0001, *t*-test for analysis. (**D**,**E**) *spen* Knockdown by 9D10 GAL4 induced the type II NBs number increase. The area outlined by the white solid line represents the region that was magnified for scanning. (**D’**) showed imINP lineages. The type II NBs were labeled with Dpn, but not with LacZ and Ase (white arrowhead) in (**D**). The white dashed circles represent type II NBs. (**E**) Quantification of type II NBs number (Ase-Dpn+ cells, white arrowhead) per brain lobe was shown in figure (**D**). **** *p* < 0.0001, Mann–Whitney Test for analysis. (**F**,**G**) Spen defect in mature INPs (mINPs) had no effect on the number of NBs. (**G**) Quantification of NBs number in each brain lobe from genotypes in (**F**), Mean ± SEM, ns, non-significant, *t*-test for analysis. GFP-marked type II NBs and their lineages in (**A**). LacZ-marked imINPs and their lineages in (**D**).

The newly generated INPs are initially immature and must prevent dedifferentiation into NBs to undergo correct differentiation into mature INPs, thereby ensuring proper brain development [16,45]. Thus, dedifferentiation of imINPs may contribute to the overproduction of type II NBs. Both imINPs and type II NBs express Pntp1(Pnt), while Dpn is only expressed in type II NBs [45,46]. We found an increase in Dpn+ pntp1+ type II NBs, while the number of imINPs (Dpn−, Pntp1+) is reduced (Figure 2A–C) with *spen* knockdown. These results indicate that the knockdown of *spen* leads to a decrease in imINPs and an increase in type II NBs. To further confirm that the *spen* knockdown leads to the dedifferentiation of imINPs into NBs, we knocked down *spen* in imINPs using the imINP-specific GAL4 (9D10) driver, and we observed that knockdown of *spen* in imINPs results in an increase in type II NBs (Dpn+ Ase−) numbers (Figure 2D,E). Furthermore, to confirm the specific role of Spen in imINPs, we knocked down *spen* using the 9D11-GAL4 (mature INPs, mINPs) driver, and found no difference in the number of type II NBs (Figure 2F,G). We conclude that Spen maintains the normal cell fate of imINPs, preventing them from dedifferentiating into type II NBs.

### 3.3. Spen Represses Notch Signaling Pathway to Prevent Overproduction of Type II NBs

**Figure 3:**
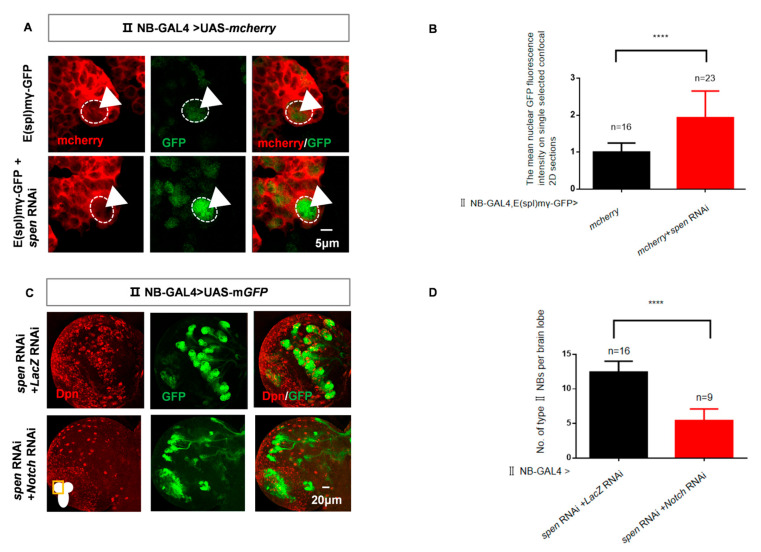
*spen* knockdown can activate the Notch signaling pathway. (**A**,**B**) The content of E(spl)mγ-GFP in *spen*-defect brains increased (white arrowhead). The white dashed circle represents type II NBs. (**B**) The mean GFP fluorescence intensity that was normalized against the background fluorescence was measured in selected regions of interest on a single selected confocal 2D section in each brain lobe from genotypes in (**A**). **** *p* < 0.0001, Mann–Whitney Test for analysis. (**C**,**D**) Double knockdown of *spen* and *notch* (B33611) rescued the increasing type II NBs number compared to knockdown of *spen* and *lacZ*. (**D**) Quantification of the number of type II NBs per brain lobe from genotypes in (**C**). Mean ± SEM, **** *p* < 0.0001, *t*-test for analysis. GFP-marked type II NBs and their lineages in (**C**); mcherry-marked type II NBs in (**A**).

Type II NBs are more sensitive to the Notch pathway [6], and activation of the Notch signaling pathway leads to an excessive number of type II NBs [19,20,21,22]. In addition, we found that the time point at which Notch activation exerted its effects was nearly identical to the time point at which *spen* knockdown exerted its effects (Figure 1I,J). Therefore, we wanted to know whether the maintenance of type II NBs by Spen was associated with Notch signaling. Firstly, we measured the expression level of downstream genes -Notch activity reporter E(spl)mγ-GFP [47] after *spen* knockdown. We found an increase in E(spl)mγ-GFP content in a single type II NB (Figure 3A,B). Next, we performed a double knockdown of *spen* and *Notch* in type II NBs. This resulted in a rescue of the type II NBs number compared to *spen* knockdown alone (Figure 3C,D). The above experimental results indicate that Spen affects the development of type II NBs through repressing the Notch signaling pathway.

Previous studies have indicated that loss of *Pnt* results in an increase in the number of type II NBs and the elimination of INPs. And the overexpression of related Notch suppressors, such as *brat* can inhibit the reversion of immature INPs to NBs [22,45,48]. To further investigate the mechanism of Spen-mediated dedifferentiation of imINPs, we first evaluated the levels of Pnt by Pnt-LacZ under *spen* knockdown. Following *spen* knockdown, the LacZ level detected in each type II NB shows no significant difference (Figure S1C,D), suggesting that *spen* deficiency did not alter *pnt* expression in individual type II NBs. Ectopic expression of *erm*, which is a downstream gene regulated by the Notch signaling pathway, can lead to the conversion of type II NBs into type I-like NBs and can also promote the termination of self-renewal in type II NBs [48]. So, we overexpressed *erm* to assess whether they could counteract the increased number of type II NBs following *spen* knockdown. We found that after overexpressing *erm* in the context of *spen* knockdown, the number of type II NBs remains approximately 13 (Figure S1E,F). Conversely, when we overexpressed *brat* with *spen* knockdown, the number of type II NBs decreased significantly (Figure S1G,H). Together, these results indicate that the dedifferentiation of imINP caused by the knockdown of *spen* can be inhibited by the Notch signaling pathway and *brat*.

### 3.4. Spen Inhibits Notch Signaling by Suppressing the Nuclear Level of NICD

**Figure 4:**
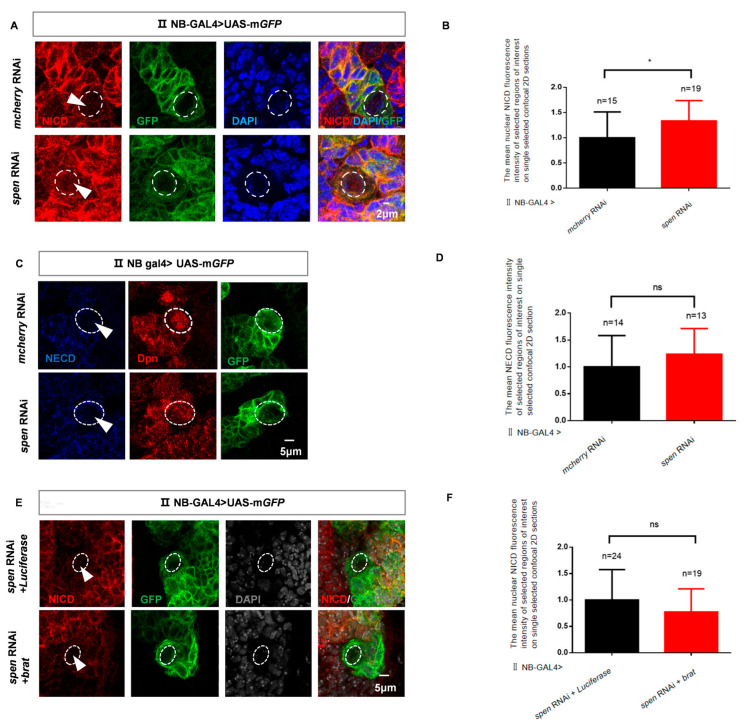
*spen* knockdown promotes nuclear nuclear Notch intracellular domain (NICD) level in Drosophila. (**A**) The mean fluorescence of NICD increased in *spen* knockdown brains. (**B**) The mean nuclear NICD fluorescence intensity that was normalized against background fluorescence was measured in selected regions of interest on a single selected confocal 2D section in each brain lobe from genotypes in (**A**). Mean ± SEM, * *p* < 0.05 T test for analysis. (**C**,**D**) The mean fluorescence of Notch extracellular domain (NECD) had no difference in *spen* knockdown brains. (**D**) The mean NECD fluorescence intensity that was normalized against background fluorescence was measured in selected regions of interest on a single selected confocal 2D section in each brain lobe from genotypes in (**C**). Mean ± SEM, ns, non-significant, *t*-test for analysis. (**E**,**F**) *Brat* overexpression in the background of *spen* knockdown could not downregulate the NICD level compared to control. (**F**) The mean nuclear NICD fluorescence intensity that was normalized against background fluorescence was measured in selected regions of interest on a single selected confocal 2D section in each brain lobe from genotypes in (**E**). Mean ± SEM, ns, non-significant, *t*-test for analysis. GFP-marked type II NBs and their lineages in (**A**,**C**,**E**). DAPI shows nuclei in (**A**,**E**). The white dashed circle represents type II NBs in (**A**,**C**,**E**).

We aimed to investigate the mechanism by which Spen represses the Notch signaling pathway, so we measured the level of Notch. Upon *spen* knockdown, we observed a moderate increase in nuclear NICD level in type II NBs (Figure 4A,B). However, the level of Notch extracellular domain (NECD) remains unchanged in type II NBs after knockdown of *spen* (Figure 4C,D). These results suggest that the content of total Notch level remains unchanged, while NICD level increases.

It had been reported that Brat could also suppress the nuclear translocation of NICD [49]. Here, our previous experimental results indicated that the overexpression of *brat* can rescue the increase in type II NBs caused by the knockdown of *spen* (Figure S1G,H). To further confirm whether the elevated nuclear NICD levels are solely due to the Spen or the result of the combined action of Spen and Brat, we overexpressed *brat* in the context of *spen* knockdown and measured the nuclear NICD level in an individual type II NB. No significant difference in nuclear NICD level is observed (Figure 4E,F). Therefore, under the condition of *spen* knockdown, Brat does not affect the increased nuclear level of NICD. The Notch inhibitor Brat can enter into imINPs from type II NBs through asymmetric division, where it suppresses the expression of downstream genes of the Notch signaling pathway, thereby preventing the dedifferentiation of imINPs [17,45]. So, these results show that in the context of imINPs, dedifferentiation is regulated by Spen. Brat may also exert its effects by downregulating the expression of Notch-related genes, rather than inhibiting the increased level of nuclear NICD induced by *spen* knockdown.

### 3.5. Hairless Promotes the Phenotype Caused by Spen in Type II NBs

**Figure 5:**
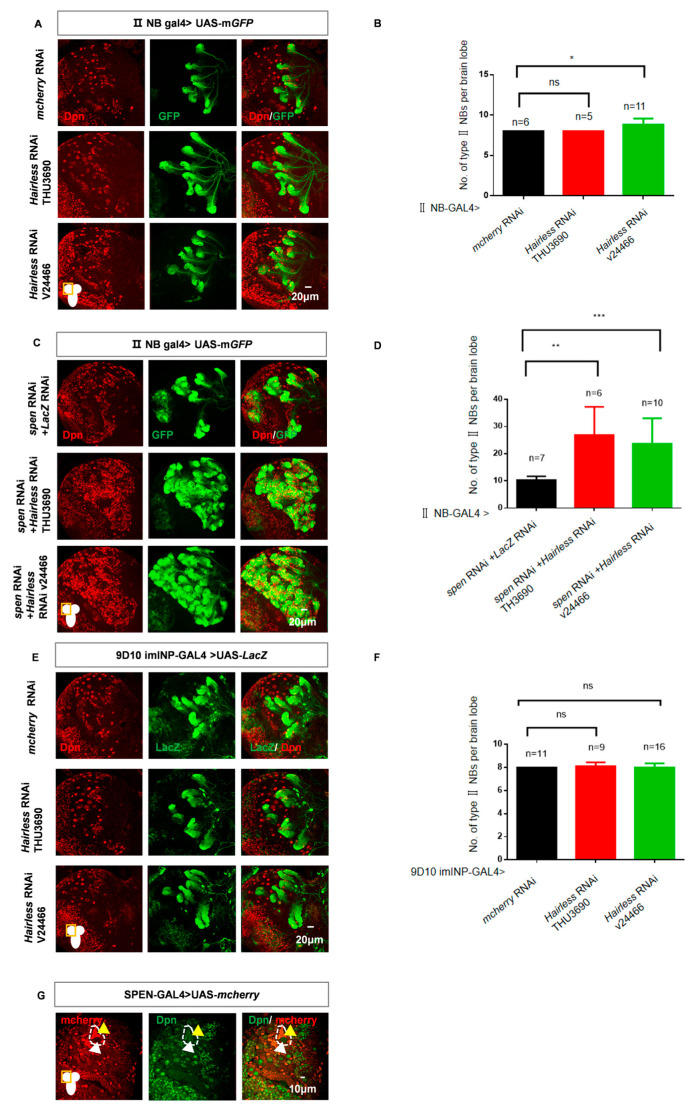
The double knockdown of *spen* and *Hairless* leads to an excessive increase in the number of type II NBs. (**A**,**B**) The number of type II NBs had a modest effect in *Hairless* knockdown brains. (**B**) Quantification of type II NBs number in per brain lobe about (**A**) * *p* < 0.05, ns, non-significant, Mann–Whitney Test for analysis. (**C**,**D**) Knockdown of *spen* and *Hairless* led to more ectopic type II NBs compared to knockdown *spen* alone. (**D**) Quantification of the number of type II NBs per brain lobe (**C**). ** *p* < 0.01, *** *p* < 0.001, Mann–Whitney Test for analysis. (**E**,**F**) *Hairless* knockdown in imINPs remained normal type II NBs number. (**F**) Quantification of type II NBs in each brain lobe from genotypes in (**E**), Mean ± SEM, ns, non-significant, *t*-test for analysis. (**G**) Spen-GAL4 derived mcherry-NLS expression. GFP-marked type II NBs and their lineages in (**A**,**C**). LacZ-marked imINPs and their lineages in (**E**). White arrowheads mark type II NBs, and yellow arrowheads mark type II NBs’ progeny.

*Hairless* is a classic gene that functions as a transcriptional inhibitor within the Notch signaling pathway in *Drosophila* [50,51]. It interacts with the CSL protein-su(H) to inhibit the Notch signaling pathway, similar to the function of SHARP in mammals [26,27]. Yet, it has been reported that Spen may not be a functional homolog of mammalian SHARP [52]. However, our experimental results suggest that Spen may serve a similar role as SHARP in type II NBs to inhibit the Notch signaling pathway. Therefore, to further investigate the function of Spen, we knocked the SHARP functional homolog gene, *Hairless,* down alone or together with *spen*. We observed a modest increase in the number of type II NBs upon knockdown of *Hairless* alone (Figure 5A,B). The effects of *Hairless* knockdown varied across different strains, which was consistent with the fact that Hairless functions in a dose-dependent manner [27]. However, a more pronounced emergence of ectopic type II NBs is noted when both *spen* and *Hairless* were simultaneously knocked down (Figure 5C,D). This indicates that both Spen and Hairless can prevent the excessive type II NBs collectively. Furthermore, since more type II NBs were observed following the knockdown of *spen* compared to the knockdown of *Hairless* alone, it appears that Spen plays a more critical role in this maintenance function than Hairless.

To further confirm that Spen and Hairless exert similar functions in the collective maintenance of type II NBs, we knocked *Ctbp* down in type II NBs alone and conducted a double knockdown experiment of *Ctbp* and *spen* simultaneously. Ctbp had been reported as another global repressor of Notch signaling pathway and recruited by Hairless in *Drosophila* or by SHARP in mammals [24,27]. We found that knockdown of *Ctbp* alone did not affect the number of type II NBs, and the double knockdown of *spen* and *Ctbp* did not exacerbate the phenotype induced by the knockdown of *spen* alone (Figure S2A–D). It suggests that in the *Drosophila* type II NBs lineage, Spen may play a similar role to Hairless, not to Ctbp.

Currently, although several co-repressors are known, the relationships between different co-repressors remain unclear; is there a tissue-specific differential involvement in their function? Our experimental results suggest that Spen may serve as an alternative co-repressor of the Notch signaling pathway in type II NBs. However, we sought to further understand the differences between Spen and Hairless as co-repressors; we knocked Hairless down in imINP and found that knockdown of Hairless in imINP does not affect the number of type II NBs, as *spen* (Figure 5E,F and Figure 2D,E). Next, we found that Spen was expressed in both type II NBs and their progeny, although its level in type II NBs appeared to be lower than in the progenitor cells (Figure 5G). Based on the above results, we concluded that Spen may play roles both in type II NBs and imINPs, whereas Hairless only exerts functions in type II NBs.

### 3.6. The EGFR Signaling Pathway Participates in the Spen-Mediated Maintenance of Type II NBs

**Figure 6:**
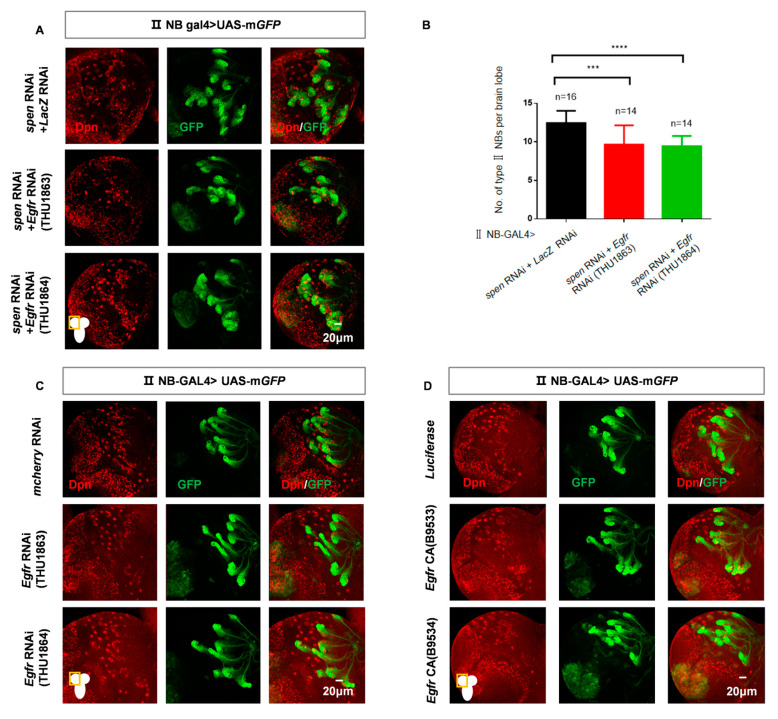
The EGFR signaling pathway is involved in Spen-mediated maintenance of type II NBs. (**A**) Double knockdown of *spen* and *Egfr* could partially rescue the increasing type II NBs number compared to knockdown of *spen* and *lacZ*. (**B**) Quantification of type II NBs number in per brain lobe from genotypes in (**A**). Mean ± SEM, *** *p* < 0.001, **** *p* < 0.0001, *t* test for analysis. (**C**,**D**) Overexpression of a constitutively active form of *Egfr* or knockdown of *Egfr* does not affect the number of type II NBs. GFP-marked type II NBs and their lineages in (**A**,**C**,**D**).

Previous research suggests that the Wnt signaling pathway (Wnt) and the EGFR signaling pathway may influence tissue development through the involvement of Spen [37,53,54,55], so we wondered whether other pathways play roles in Spen-mediated maintenance of type II NBs. We found that the knockdown of *Arm*, a core component of the *Drosophila* Wnt signaling pathway and homolog of β-catenin, failed to change the effects in *spen* knockdown background (Figure S2E). Reducing the activity of the EGFR pathway could partially decrease the type II NBs in the *spen* knockdown background (Figure 6A,B). However, direct knockdown of *Egfr* in type II NBs or expression of a constitutive active form of *Egfr* does not affect the type II NBs (Figure 6C,D). The above results demonstrate that the EGFR signaling pathway is involved in the Spen-mediated maintenance of type II NBs, rather than independently regulating type II NBs during the third larval stage.

## 4. Discussion

**Figure 7:**
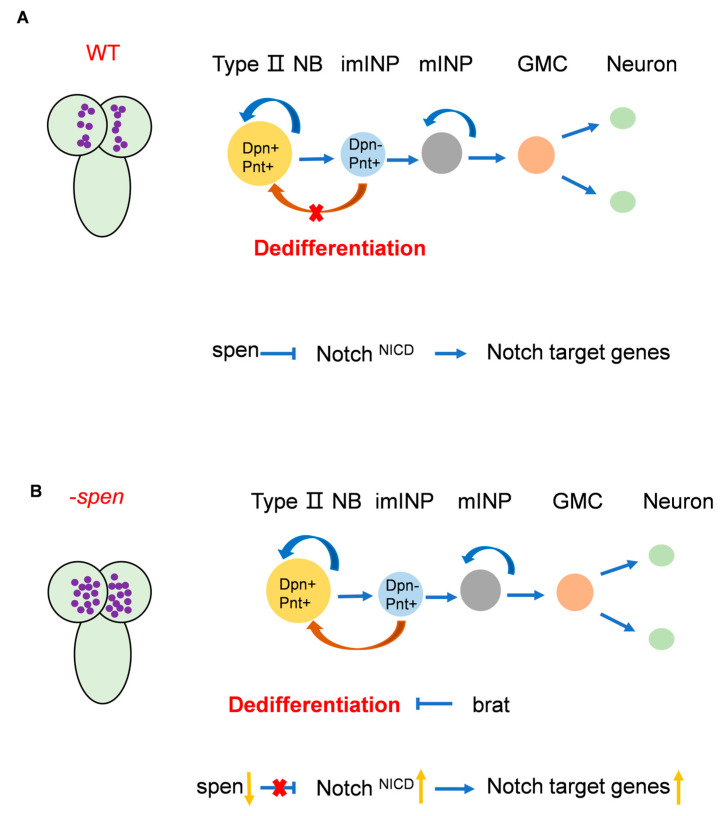
Pattern diagram of the role of Spen in type II NBs. (**A**) In wildtype, Spen may act as a co-repressor to regulate nuclear NICD levels, thereby repressing the expression of genes downstream of the Notch signaling pathway. So that the number of type II NBs can be maintained at normal levels. (**B**) In the absence of Spen, the level of nuclear NICD is elevated, resulting in increased expression of Notch signaling pathway genes. Then imINPs dedifferentiate into type II NBs to increase the number of type II NBs. Brat can inhibit this dedifferentiation.

Different neural stem cells produce varying numbers of progeny cells to maintain normal brain development, making it crucial to investigate the factors that regulate the development of distinct neural stem cell populations. Given that the progeny production pattern of *Drosophila* type II NBs is similar to that of higher mammals, investigating new factors involved in maintaining *Drosophila* type II NBs could provide insights for future studies on the maintenance of neural stem cells in higher mammals. Although factors regulating different NBs in *Drosophila* have been reported, it remains unclear whether there are additional and important factors yet to be discovered. In this article, we found that *spen* knockdown leads to an increase in the number of type II NBs by inhibiting the Notch^NICD^ level to prevent the dedifferentiation of imINPs (Figure 7). This phenotype specifically occurs in type II NBs and is not present in type I NBs, neurons, or glial cells. A recent study by Li et al., published on bioRxiv, similarly showed that the loss of Spen results in elevated expression of E(spl)m γ in stem cells, which in turn induces dedifferentiation in their descendant cells. These findings collectively support our own experimental conclusions. Additionally, we aim to investigate whether Spen functions as a co-repressor regulating nuclear NICD to control the expression of downstream genes such as E(spl)m γ. However, Li et al. found that Spen inhibits the translation of E(spl)m γ in stem cells by directly interacting with conserved motifs to keep E(spl)m γ expression at a low level. These findings indicate that Spen may regulate the Notch signaling pathway in type II NBs through multiple mechanisms, further highlighting the crucial role of Spen in *Drosophila* neural stem cell development [56]. Although Spen has been investigated in various *Drosophila* tissues, including the eyes, intestinal stem cells, and glial cells [32,37,41], its specific function in *Drosophila* NBs has remained unclear. Our findings, together with those of Li et al., provide additional insight into the role of Spen in the maintenance of type II NBs.

In *Drosophila*, Hairless is known as a co-repressor of the Notch signaling pathway, recruiting factors such as CtBP to collectively inhibit Notch signaling [27,57]. However, in mammals, there is no homolog of Hairless, and thus this process is carried out by the *Drosophila* Spen homolog, SHARP [24,52]. Some studies have suggested that *Drosophila* Spen may not functionally correspond to mammalian SHARP [52]. Our experimental results indicate that the concomitant knockdown of *spen* and *Hairless* results in a greater increase in type II NBs compared to the individual knockdown of either *spen* or *Hairless*. Furthermore, the phenotype resulting from the knockdown of *spen* is noticeably more pronounced than that from the knockdown of *Hairless*, suggesting that Spen may play a more critical role than Hairless. Based on our findings regarding the mechanism by which Spen inhibits Notch signaling, we propose that Spen and Hairless may function together as co-repressors. While the binding of mammalian SHARP to RBP-J has been reported, the interaction of Spen as a co-repressor with the *Drosophila* RBP-J homolog su(H) also requires investigation. This will be the focus of our future work, as we aim to provide more definitive evidence for the existence of two distinct co-repressors in *Drosophila* type II NBs. The presence of these two different co-repressors within the same lineage raises questions about their functional roles—possibly exerting different effects in distinct cell types? Our research indicates that the knockdown of *spen* in type II NBs or imINPs leads to a specific increase in type II NBs, while Hairless appears to function solely within type II NBs. This phenotype may be due to the fact that Spen and Hairless regulate distinct Notch downstream target genes, which is another avenue for our future exploration. In addition, it will be an intriguing area of future research to investigate when Hairless begins to disappear in various species and how its function is gradually replaced by SHARP.

Spen has been reported to influence diseases by modulating signaling pathways. For instance, Spen can regulate nasopharyngeal carcinoma (NPC) by maintaining the levels of PI3K/AKT and c-JUN [40]. And in *Drosophila* tissues, different signaling pathways often work together to regulate development. Our study shows that during the third instar larval stage, not only is the Notch signaling pathway crucial for the development of type II NBs, but the Egfr signaling pathway also plays a role in maintaining the number of NBs. This effect is mediated exclusively by Spen. Studying the regulation of type II NBs development by different signaling pathways is crucial for maintaining the number of type II NBs. However, the mechanisms by which these two signaling pathways collaboratively regulate each other remain unclear. In the future, we will pursue this as our research objective, aiming to target in order to specifically modulate the interactions between different signaling pathways, with the goal of addressing related diseases.

## Data Availability

The original contributions presented in this study are included in the article/Supplementary Material. Further inquiries can be directed to the corresponding author.

## Supplementary Materials

The following supporting information can be downloaded at: https://www.mdpi.com/article/10.3390/cells14231926/s1, Figure S1. (A) Mira retains at the basal cortex of the NBs (indicated by the white arrowheads in the first and second rows) and aPKC located at apical cortex of NBs (Indicated by the white arrowheads in the third and fourth rows) when *spen* knockdown. (B) Quantification of aPKC and Mira localization during metaphase from genotypes in (A). (C,D) Knockdown of *spen* had no impact of the level of Pnt protein in an individual type II NB. (D) The mean LacZ fluorescence intensity that was normalized against background fluorescence was measured in selected regions of interest on single selected confocal 2D sections in per brain lobe from genotypes in (C). Mean SEM, ns, non significant, T test for analysis. (E,F) The overexpression of *erm* in the background of *spen* knockdown preserved the phenotype of increased type II NB numbers. (E) Quantification of type II NBs number in per brain lobe from genotypes in (D), Mean SEM, ns, non significant, T test for analysis. (G,H) Overexpression of *brat* decreased the number of type II NBs. Type II NB lineages were labeled by GFP, Dpn, and Ase (white arrowheads). (H) Quantification of type II NBs number in per brain lobe from genotypes in (G). **** *p* < 0.0001, Mann Whitney Test for analysis. GFP marked type II NBs and their lineages in A, C, E and G; Figure S2. (A,B) Knockdown of *Ctbp* could not led to the change of type II NBs number. (B) Quantification of type II NBs number in per brain lobe from genotypes in (A). Mean ± SEM, ns, non significant, T test for analysis. (C,D) knockdown of *spen* and *Ctbp* did not affect the number of type II NBs compared to *spen* knockdown alone. (D) Quantification of type II NBs number in per brain lobe from genotypes in (C). Mean ± SEM, ns, non significant, T test for analysis. (E) Downregulation of the Wingless signaling pathway did not affect the increase in the number of type II NBs caused by *spen* knockdown. GFP marked type II NBs and their lineages in A, C and E; Table S1. The exact *p* values in the article.
