# Supplementary material for: Split Ends Inhibits the Dedifferentiation of imINP to Prevent the Generation of Supernumerary Type II Neuroblasts in Drosophila
Source: Cells. 2025 Dec 4;14(23):1926. doi: 10.3390/cells14231926 (PMC12691262; doi:10.3390/cells14231926)
Supplement: Supplementary file 1 [file cells-14-01926-s001.zip › cells-4000625-supplementary.pdf]

Supplementary Materials:

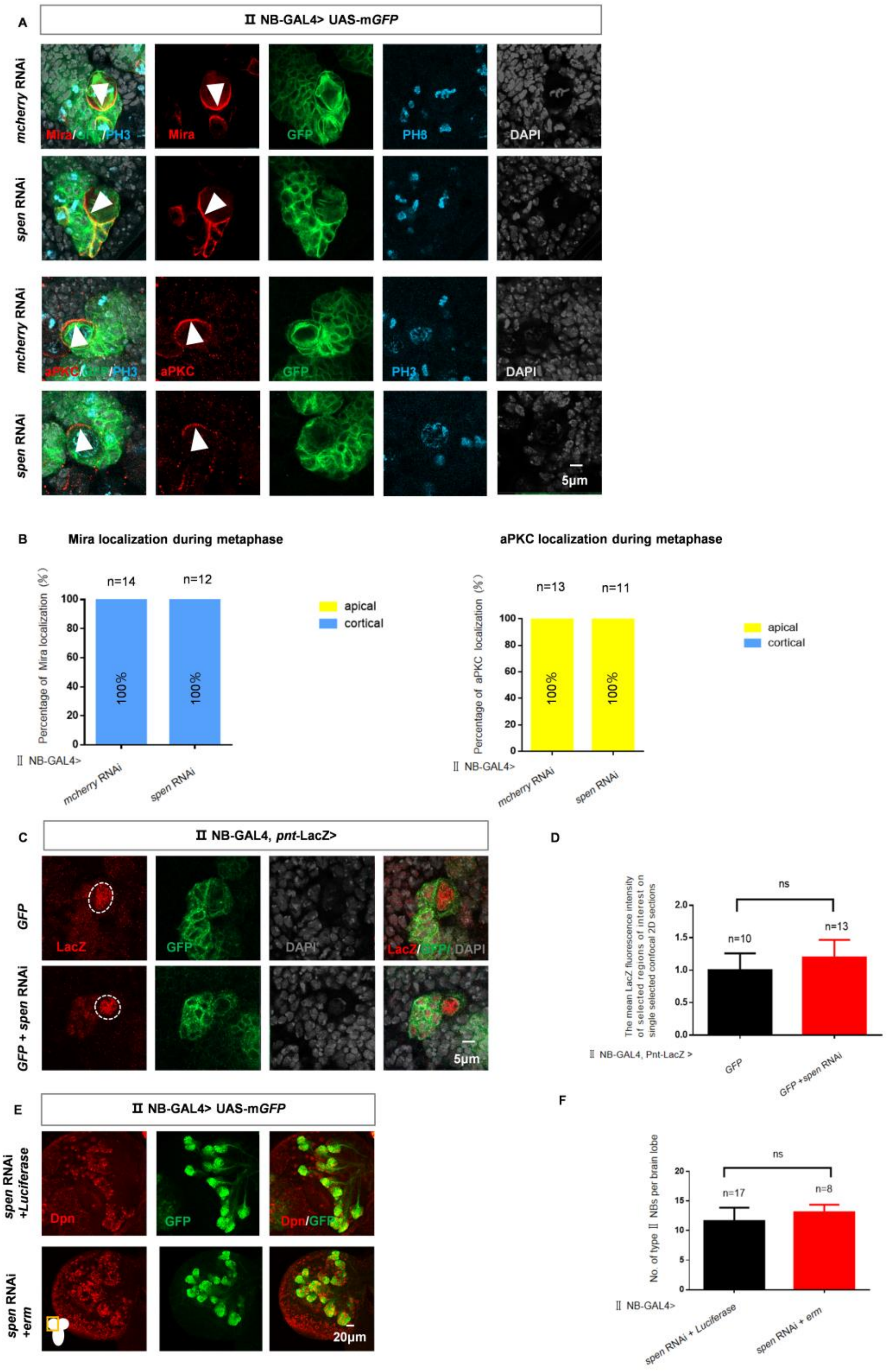

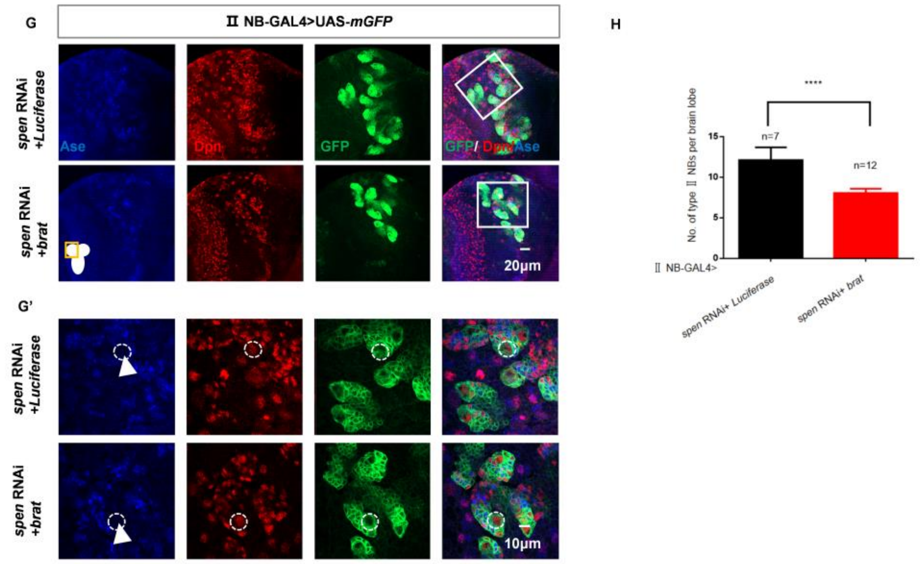

Figure S1. (A) Mira retains at the basal cortex of the NBs (indicated by the white arrowheads in the first and second rows) and aPKC located at apical cortex of NBs (Indicated by the white arrowheads in the third and fourth rows) when *spen* knockdown. **(B)** Quantification of aPKC and Mira localization during metaphase from genotypes in (A).**(C-D)** Knockdown of *spen* had no impact of the level of Pnt protein in an individual type II NB. **(D)** The mean LacZ fluorescence intensity that was normalized against background fluorescence was measured in selected regions of interest on single selected confocal 2D sections in per brain lobe from genotypes in (C). Mean ± SEM, ns, non-significant, T test for analysis. **(E-F)** The overexpression of *erm* in the background of *spen* knockdown preserved the phenotype of increased type II NB numbers. (E) Quantification of type II NBs number in per brain lobe from genotypes in (D), Mean ± SEM, ns, non-significant, T test for analysis. **(G-H)** Overexpression of *brat* decreased the number of type II NBs. Type II NB lineages were labeled by GFP, Dpn, and Ase (white arrowheads). **(H)** Quantification of type II NBs number in per brain lobe from genotypes in (G). ****P<0.0001, Mann-Whitney Test for analysis. GFP marked type II NBs and their lineages in A , C, E and G.

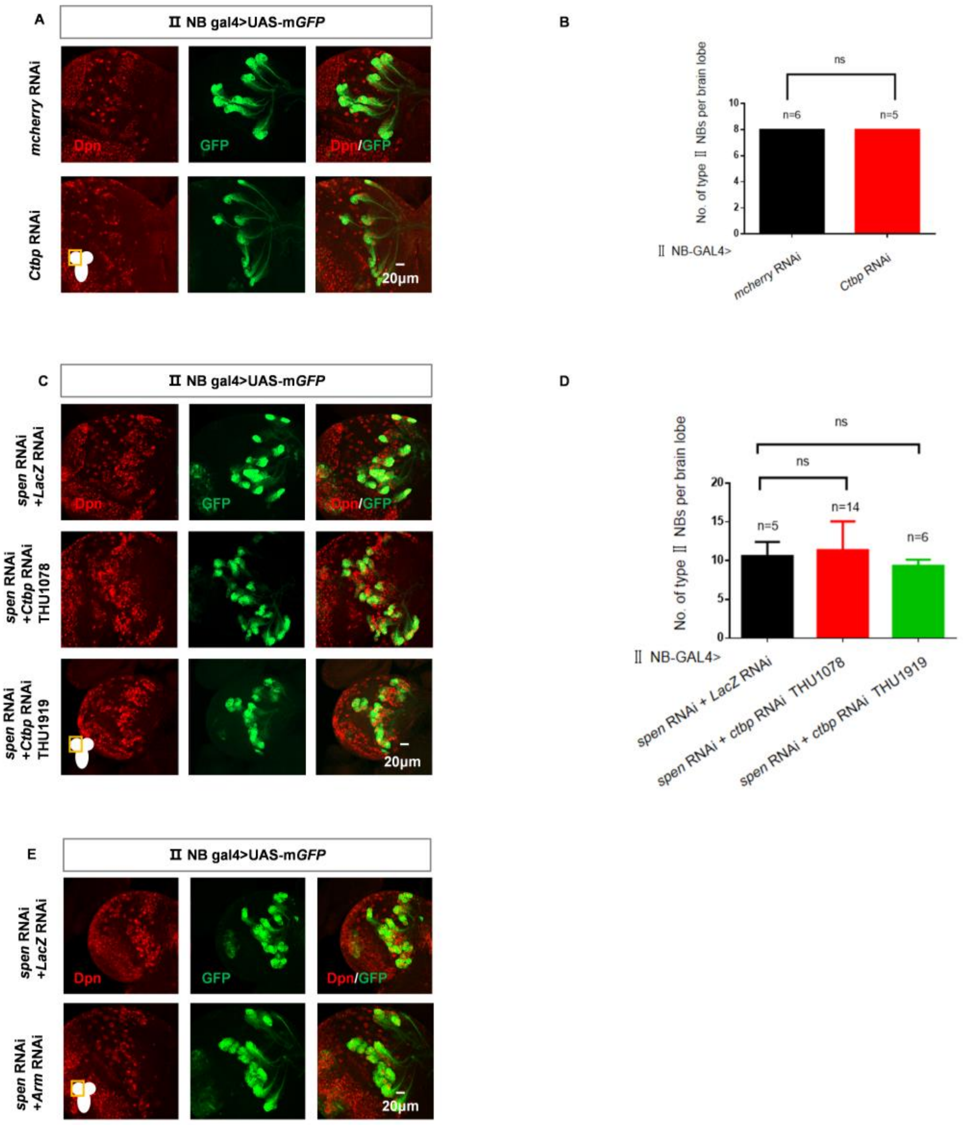

Figure S2. **(A-B)** Knockdown of *Ctbp* could not led to the change of type II NBs number. **(B)** Quantification of type II NBs number in per brain lobe from genotypes in (A). Mean ± SEM, ns, non-significant, T test for analysis. **(C-D)** knockdown of *spen* and *Ctbp* did not affect the number of type II NBs compared to *spen* knockdown alone. **(D)** Quantification of type II NBs number in per brain lobe from genotypes in (C). Mean ± SEM, ns, non-significant, T test for analysis. **(E)** Downregulation of the Wingless signaling pathway did not affect the increase in the number of type II NBs caused by *spen* knockdown. GFP marked type II NBs and their lineages in A, C and E.

Table S1. The exact p-values in the article

Figure	p-value	*
F1B THU0750	0.0000034022	****
F1B v48846	0.0000680434	****
F1B v108828	0.0000578369	****
F1E	0.0101000000	*
F2B	0.0000075159	****
F2C	0.0000000027	****
F2E	0.0000004895	****
F3B	0.0000000485	****
F3D	0.0000000004	****
F4B	0.0393263031	*
F4D	0.2617221417	ns
F4F	0.1631894412	ns
F5B v24466	0.0256140918	*
F5D THU3690	0.0012000000	**
F5D v24466	0.0003000000	***
F6B THU1863	0.0009000000	***
F6B THU1864	0.0000061198	****
Fs1D	0.0904000000	ns
Fs1F	0.0961000000	ns
Fs1H	0.0000198460	****
Fs2D THU1078	0.6712000000	ns
Fs2D THU1919	0.1571000000	ns
